# Tunable multichannel Photonic spin Hall effect in metal-dielectric-metal waveguide

**DOI:** 10.1038/s41598-021-93517-w

**Published:** 2021-07-08

**Authors:** Li-Ming Zhao, Yun-Song Zhou

**Affiliations:** Department of Physics, Capital Normal University, Beijing, 100048 China

**Keywords:** Optical materials and structures, Nanophotonics and plasmonics

## Abstract

The discovery of Photonic spin Hall effect (PSHE) on surface plasmon polaritons (SPPs) is an important progress in photonics. In this paper, a method of realizing multi-channel PSHE in two-dimensional metal-air-metal waveguide is proposed. By modulating the phase difference $\phi$ and polar angle $\theta$ of the dipole source, the SPP can propagate along a specific channel. We further prove that PSHE results from the component wave interference theory. We believe that our findings will rich the application of SPPs in optical devices.

## Introduction

Surface plasmon polaritons (SPPs) have attracted enormous attention because it can overcome the diffraction limits of light and are easy to manipulation on subwavelength scale^1,2^. Therefore, many applications on the basis of surface plasmon nanostructures have been proposed such as beam splitters^3^, nano-sensors^4^, switches^5,6^, color filters^7^, perfect absorbers^8^ and so on. Photonic spin Hall effect (PSHE) in SPP system^9,10^ provides a convenient way to control the direction of light propagation by using the incident polarization state and this fascinating behavior results from the spin-momentum locking of SPP, which rigidly connects the direction of propagation of the SPP wave with its transverse spin angular momentum^11–22^. One of the explanations about PSHE are the spin coupling theories^11^. It’s thought that the excited SPP should have the same spin with that of incident beam, and then the SPP propagation direction can be determined according to the chirality. In fact, the angular momentum is not conserved when the incident light is converted into SPP waves. Recently, it is found that the asymmetric scattering of PSHE results from the component wave interference (CWI) of electric or magnetic dipoles^9,23,24^, and this interference effect of the elliptical polarized dipole results in the selective directional scattering.

The realization and application of surface plasma waveguide is of great significance because it is key to realize all-optical circuit, and it possesses unique advantages such as strong confinement, low bend loss and so on. Based on the understanding of component wave interference of incident light (CWI theory)^23,24^, we believe PSHE can be realized in the waveguide structure. Motivated by this consideration, we hope to realize PSHE in metal-air-metal plasmatic waveguides, which will be greater progress towards practical applications. In this paper, we achieve tunable multi-tunnel PSHE in two-dimensional metal-air-metal waveguides, we believe that theses results will provide significant applications in optical devices.

## Results and analysis

**Figure 1:**
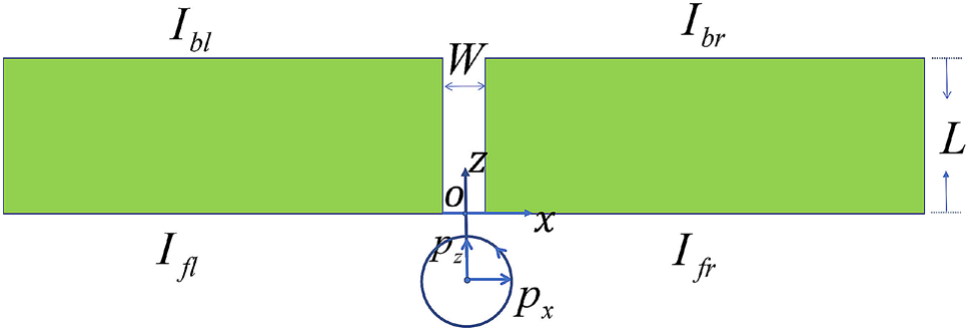
Sketch map of metal-air-metal waveguide, the width of the waveguide is *W* and length is *L*. There are four interfaces named by $I_{bl}$, $I_{br}$, $I_{fl}$, $I_{fr}$.

We build a silver-air-silver waveguide as shown in Fig. 1, the width of the waveguide is *W* and the length is *L*. The coordinate system was builded as Fig. 1. We assume a dipole source $\vec{p}=(cos\theta \hat{x}+sin \theta e^{i\phi }\hat{z})e^{-i\omega t}$ is located at the position of $x=0$ and $z=z_0$, here $\phi$ is referred to **phase difference** of the dipole source, $\theta$ is called **polar angle**. The radiation wavelength is $0.6 \upmu {\text{m}}$. Therefore, the corresponding dielectric constant of *Ag* is $\epsilon =-15.04+i1.01$ for this wavelength. It is obvious that the SPP may propagate along the four interfaces, we called the four interfaces as $I_{bl}$, $I_{br}$, $I_{fl}$, $I_{fr}$, as shown in Fig. 1. In this paper, the finite-difference time-domain (FDTD) method with the perfectly matched layer (PML) is used to study the electromagnetic field in the construction.

**Figure 2:**
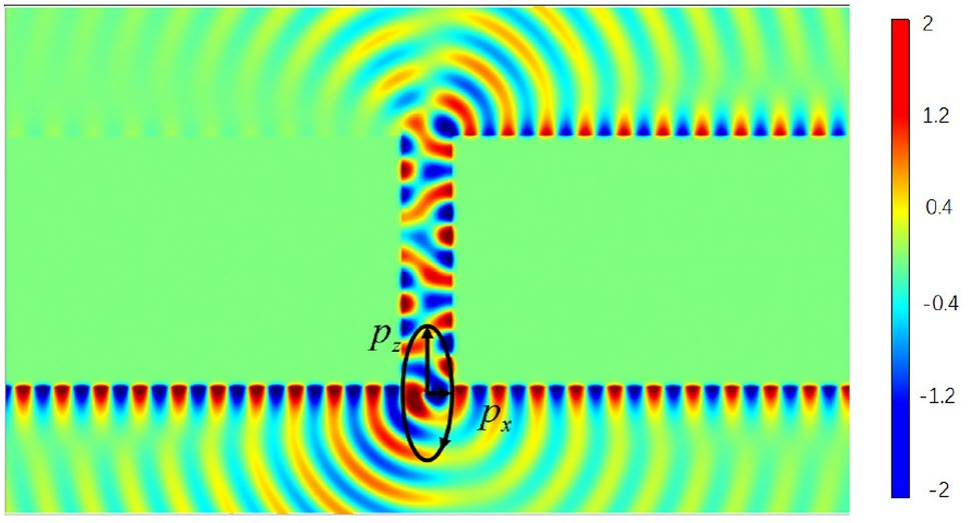
Distribution of field in the waveguide, and the waveguide is with $W=1.2 \upmu {\text{m}}$ and $L=6 \, \upmu {\text{m}}$, the dipole source $\vec{p}=(cos70^{\circ } \hat{x}+sin 70^{\circ } e^{i 130^{\circ }}\hat{z})e^{-i\omega t}$, which is located at $x=0$ and $z_0 =-0.1 \, \upmu {\text{m}}$.

Figure 2 shows the field distribution in the case of $z_0 =-0.1 \upmu {\text{m}}$ for $\vec{p}=(cos70^{\circ } \hat{x}+sin 70^{\circ } e^{i 130^{\circ }}\hat{z})e^{-i\omega t}$. The waveguide width is $W=1.2 \upmu {\text{m}}$ and the length is $L=6 \, \upmu {\text{m}}$. We find that SPP is mainly focused on the three interfaces, and for the interface $I_{bl}$, the energy of SPP is nearly zero. Therefore, the PSHE can be achieved successfully in this configuration. Now, we first pay attention to the back interface, including the interfaces $I_{bl}$ and $I_{br}$. In order to quantify the degree of asymmetric propagation of SPP, we define the separation degree $\eta _{b}=\frac{P_{br}}{|P_{br}|+|P_{bl}|}$, here, $P_{br}$ is the total power for SPP at the interface $I_{br}$, and $P_{bl}$ is the total power for SPP at the interface $I_{bl}$. When $\eta =0.5$, the energies of the SPP propagating along the $I_{br}$ and $I_{bl}$ are the same, $\eta >0.5$, SPP propagates mainly along $I_{br}$, and $\eta <0.5$, the SPP propagates mainly along $I_{bl}$. In the same way, we can also define $\eta _{f}$, which represents the asymmetric propagation characteristic of SPP along the interfaces $I_{fl}$ and $I_{fr}$ (front interface). In Fig. 2, $\eta _{b} =0.9962$ and $\eta _{f} =0.3896$, the corresponding SPP propagates mainly to the right for the back interface and to the left for the front interface. We further find that the energy is mainly focused on the front interface, The total energy ratio of the front and back interface is about 5:1.

**Figure 3:**
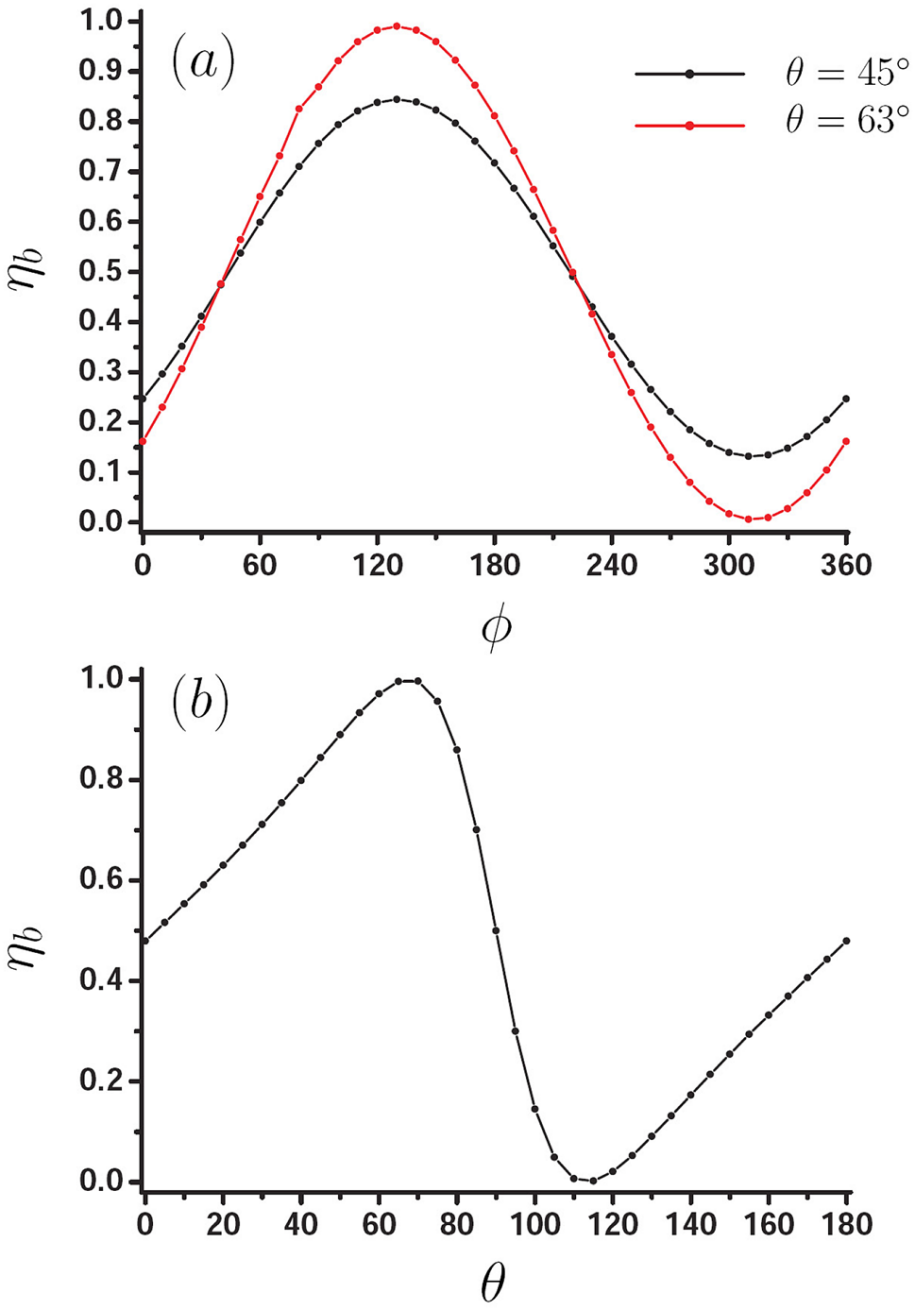
Effect of $\phi$ and $\theta$ on PSHE for the waveguide with $W=1.2 \, \upmu {\text{m}}$ and $L=6 \, \upmu {\text{m}}$, the dipole source is located at $x=0$, and $z_0 =-0.1 \, \upmu {\text{m}}$. (**a**) Separation degree $\eta _{b}$ as a function of $\phi$ for the two different $\theta$, black curve for $\theta =45^{\circ }$ and red curve for $\theta =63^{\circ }$. (**b**) Separation degree $\eta _{b}$ as a function of $\theta$ when $\phi =130^{\circ }$.

Now, we investigate the effect of phase difference $\phi$ and polar angle $\theta$ on PSHE. We adopt the same parameters as Fig. 2, that is, the width and length of the waveguide are $W=1.2 \, \upmu {\text{m}}$ and $L=6\, \upmu {\text{m}}$. Figure 3a shows separation degree $\eta _{b}$ as a function of $\phi$ for the two different $\theta$, black curve for $\theta =45^{\circ }$ and red curve for $\theta =63^{\circ }$. It is obvious that the two curves present the same oscillating behavior, and the optimal separation degree is located at $\phi =130^{\circ }$. Figure 3b presents $\eta _{b}$ as a function of $\theta$ for $\phi =130^{\circ }$. The optimal PSHE is about $\theta =70^{\circ }$ and $\theta =115^{\circ }$, the corresponding separation degree is about 0.996 and 0.0002 respectively. These results further denote that the PSHE can be well modulated by $\phi$ and $\theta$ of dipole source.

**Figure 4:**
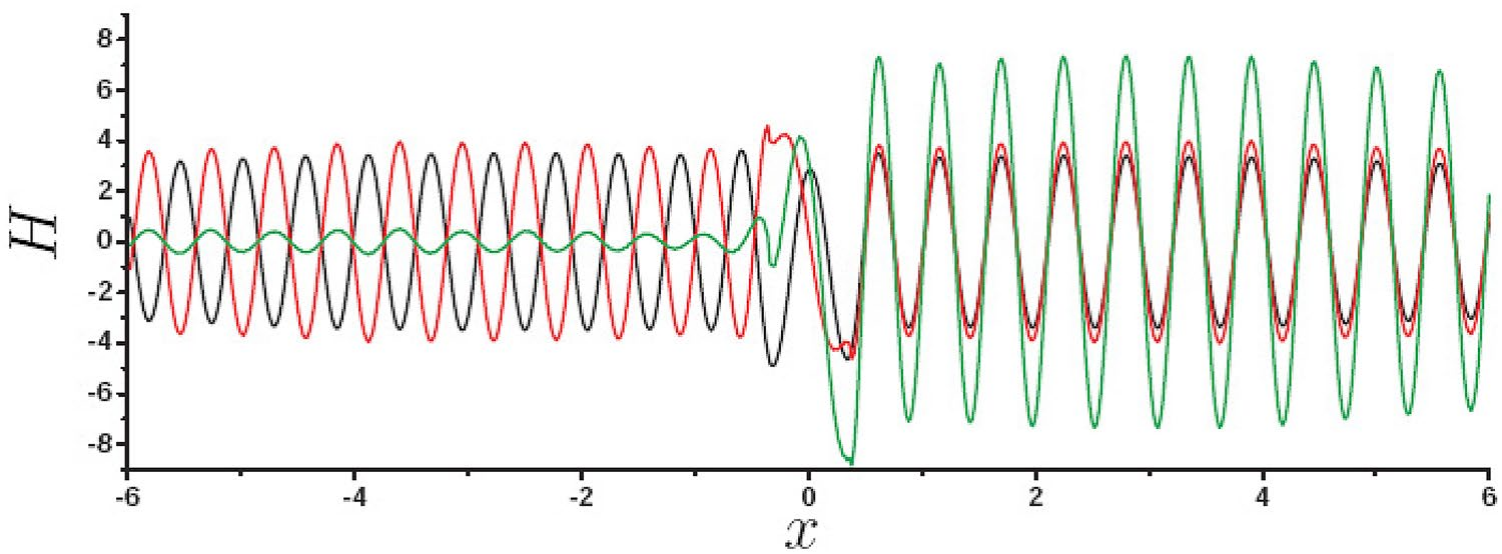
Surface field at $z=6\, \upmu {\text{m}}$ as a function of *x* for different $\vec{p}$, black curve for $\vec{p}=cos70^{\circ }e^{-i\omega t} \hat{x}$, red curve for $\vec{p}=sin 70^{\circ } e^{i130^{\circ }} \hat{z}e^{-i\omega t}$, and green curve for $\vec{p}=(cos70^{\circ } \hat{x}+sin 70^{\circ }e^{i130^{\circ }} \hat{z})e^{-i\omega t}$.

In order to understand how $\phi$ and $\theta$ affect PSHE, Fig. 4 shows the distribution of field at the back interface for different dipole sources, black curve for $\vec{p}=cos70^{\circ } \hat{x}e^{-i\omega t}$, red curve for $\vec{p}=sin 70^{\circ }e^{i130^{\circ }} \hat{z}e^{-i\omega t}$, green curve for $\vec{p}=(cos70^{\circ } \hat{x}+sin 70^{\circ }e^{i130^{\circ }} \hat{z})e^{-i\omega t}$. The results show that the field stimulated by $p_x$ is an even function about $x=0$, and the field stimulated by $p_z$ is an odd function about $x=0$, and the total field for the green curve is the coherent superposition of black curve and red curve. Obviously, green curve shows the asymmetric propagation of SPP. Therefore, the PSHE results from the superposition of fields with different parity excited by $p_x$ and $p_z$, this is so called CWI theory. If the two kind of SPPs at the back interface (black curve and red curve in Fig. 4) have the same amplitude and phase, the PSHE is strong, we further find that the phase is dominated by $\phi$ and amplitude is determined by $\theta$. Therefore, we can obtained the optimal PSHE by modulating $\theta$ and $\phi$. Here, it should be emphasized that in CWI theory, the main scattering direction of SPP is determined by $\phi$ (the incident spin). Since the spin and orbit of SPP are locked, the scattering direction determines the spin of SPP.

It is worth noting that there are odd and even waveguide modes in this metal-air-metal waveguide. The odd waveguide modes can be excited by z-oriented dipole $p_{z}$ and even waveguide modes can be excited by x-oriented dipole $p_{x}$. The wave vectors of the odd and even waveguide modes are different along the $z-$direction, this can bring the phase difference in the waveguide. Therefore, the phase difference of the SPPs at the back interface are determined by the following two reasons, one is the waveguide length *L*, the other is $\phi$ of the diploe source.

**Figure 5:**
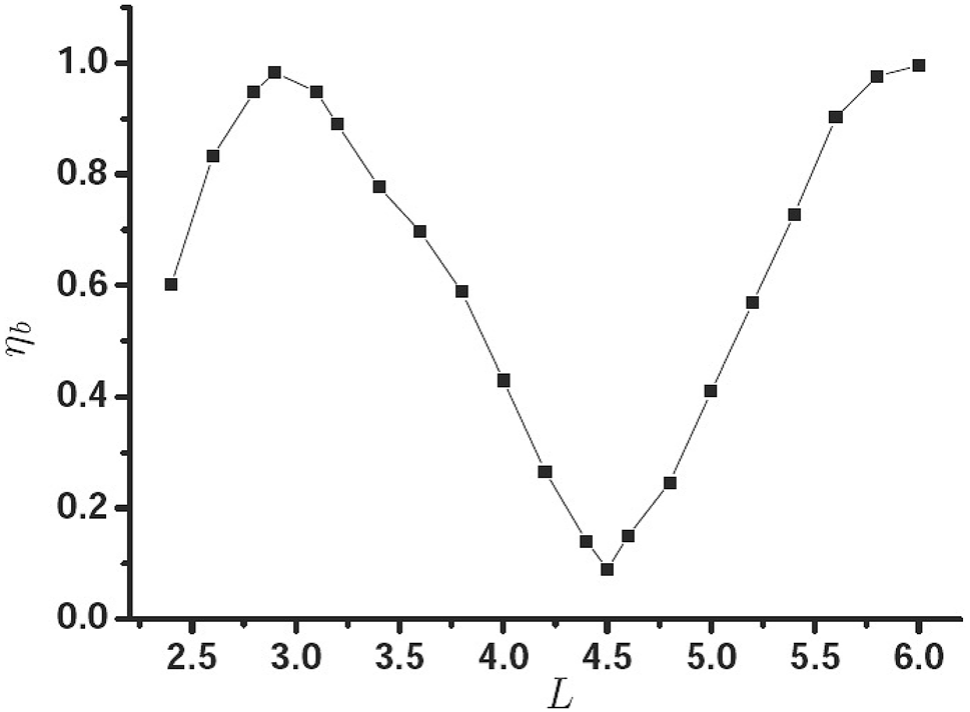
$\eta _{b}$ as a function of *L*.

In order to prove this point, Fig. 5 gives $\eta _{b}$ as a function of *L*, the other parameters are the same with Fig. 2. It is clearly seen that the $\eta _{b}$ oscillates with the length of waveguide *L*. When $L=2.9\, \upmu {\text{m}}$, $4.5 \, \upmu {\text{m}}$, and $6.0 \, \upmu {\text{m}}$, the PSHE is the optimal.

**Figure 6:**
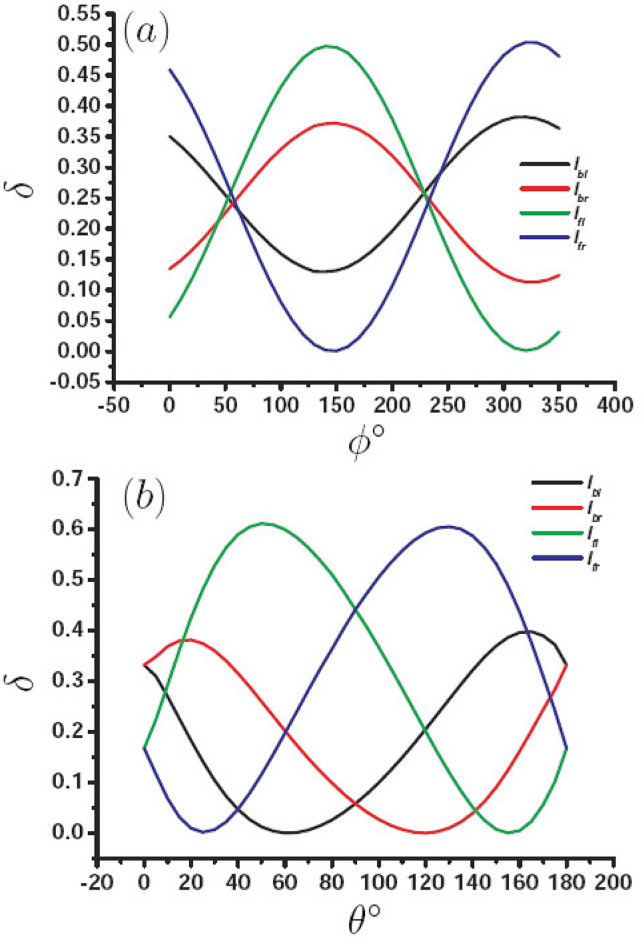
Energy proportion $\delta$ for four different interface modes as a function of $\phi$ and $\theta$. (**a**) variation of $\delta$ with $\phi$, (**b**) variation of $\delta$ with $\theta$.

Now, we would like to achieve tunable PSHE for the four interfaces (four channels) by modulating $\theta$ and $\phi$. In order to quantitatively describe the energy proportion in each channel, we define the energy proportion $\delta$. As an example, for $I_{bl}$ channel, $\delta _{bl} =\frac{P_{bl}}{P_{t}}$, here, $P_{t}=P_{bl}+P_{br}+P_{fl}+P_{fr}$ is the total energy. Fig. 6 describes the energy proportion as a function of $\phi$ and $\theta$ for $L=3\, \upmu {\text{m}}$, (a) $\delta$ as a function of $\phi$ when $\theta =27^{\circ }$, (b) $\delta$ as a function of $\theta$ when $\phi =140^{\circ }$, black curve represents the $I_{bl}$ channel, red curve is the $I_{br}$ channel, green curve is the $I_{fl}$ channel, and blue curve for the $I_{fr}$ channel. For Fig. 6a, it can be found that when $-130^{\circ }\le \phi < 50^{\circ }$, the energy mainly focused on $I_{bl}$ and $I_{fr}$ channels. When $50^{\circ }< \phi < 230^{\circ }$, the energy is mainly focused on $I_{br}$ and $I_{fl}$ channels. When $\phi =50^{\circ }$ and $\phi =230^{\circ }$, the energy in the four channels is equal. For Fig. 6b, it can be find that the energy is focused on the front interfaces when $20^{\circ }<\theta < 160^{\circ }$, but the energy proportion can be modulated by $\theta$. In order to exhibit clearly the modulation of $\theta$ and $\phi$ on the energy proportion in the four channels, Stable I gives the energy proportion for different $\theta$ and $\phi$, that can clearly tell us in which channels the SPPs propagate along.

It can be concluded from Table 1 that we can achieve SPP concentration on two channels, for example $\theta =27^{\circ }$ and $\phi =150^{\circ }$, and energy concentration on $I _{br}$ and $I _{fl}$; we can also make SPP disappear in one channel for example, $\theta =60^{\circ }$ and $\phi =140^{\circ }$, and SPP disappear in $I _{bl}$ channel; SPP can also have the nearly same energy in the four channels, for example, $\theta =27^{\circ }$ and $\phi =50^{\circ }$. We believe that the flexible modulation of SPP in the channels will bring significant application prospect in the optical devices.

**Table 1:** Energy proportion for different $\theta$ and $\phi$.

$\theta$	$\phi$	$\delta _{bl}$	$\delta _{br}$	$\delta _{fl}$	$\delta _{fr}$
$27^{\circ }$	$50^{\circ }$	0.255	0.226	0.239	0.280
$27^{\circ }$	$150^{\circ }$	0.132	0.372	0.496	0.0007
$27^{\circ }$	$330^{\circ }$	0.379	0.113	0.005	0.503
$0^{\circ }$	$140^{\circ }$	0.332	0.332	0.168	0.168
$10^{\circ }$	$140^{\circ }$	0.270	0.369	0.293	0.068
$60^{\circ }$	$140^{\circ }$	0.0002	0.202	0.600	0.197
$85^{\circ }$	$140^{\circ }$	0.041	0.077	0.478	0.404
$120^{\circ }$	$140^{\circ }$	0.206	0.00003	0.201	0.594
$170^{\circ }$	$140^{\circ }$	0.390	0.244	0.056	0.310

## Summary

The asymmetric scattering mechanism of PSHE in two-dimensional metal waveguide is studied, and we further prove that this is caused by the CWI theory, that is, PSHE is caused by the interference of the component SPPs excited by the different components of the incident light. The phase difference of two different SPPs is dominated by $\phi$ of the dipole source, and the amplitudes is determined by $\theta$ of the dipole source. We further find that a tunable multi-channel PSHE can realized by by modulating $\theta$ and $\phi$.
